# Regional Heritability Mapping Provides Insights into Dry Matter Content in African White and Yellow Cassava Populations

**DOI:** 10.3835/plantgenome2017.06.0050

**Published:** 2017-12-22

**Authors:** Uche Godfrey Okeke, Deniz Akdemir, Ismail Rabbi, Peter Kulakow, Jean-Luc Jannink

**Affiliations:** 1 Section of Plant Breeding and Genetics, School of Integrative Plant Sci., College of Agriculture and Life Sci., Cornell Univ., 14853, Ithaca, NY; 2 current address, Statgen Consulting, Ithaca, NY 14850; 3 IITA, PMB 5320, Oyo Road, Ibadan, Nigeria; 4 USDAARS, Robert W. Holley Centre for Agriculture and Health, Tower Road, Ithaca, NY 14853

## Abstract

The HarvestPlus program for cassava (*Manihot esculenta* Crantz) fortifies cassava with β-carotene by breeding for carotene-rich tubers (yellow cassava). However, a negative correlation between yellowness and dry matter (DM) content has been identified. We investigated the genetic control of DM in white and yellow cassava. We used regional heritability mapping (RHM) to associate DM with genomic segments in both subpopulations. Significant segments were subjected to candidate gene analysis and candidates were validated with prediction accuracies. The RHM procedure was validated via a simulation approach and revealed significant hits for white cassava on chromosomes 1, 4, 5, 10, 17, and 18, whereas hits for the yellow were on chromosome 1. Candidate gene analysis revealed genes in the carbohydrate biosynthesis pathway including plant serine–threonine protein kinases (SnRKs), UDP (uridine diphosphate)-glycosyltransferases, UDP-sugar transporters, invertases, pectinases, and regulons. Validation using 1252 unique identifiers from the SnRK gene family genome-wide recovered 50% of the predictive accuracy of whole-genome single nucleotide polymorphisms for DM, whereas validation using 53 likely genes (extracted from the literature) from significant segments recovered 32%. Genes including an acid invertase, a neutral or alkaline invertase, and a glucose-6-phosphate isomerase were validated on the basis of an a priori list for the cassava starch pathway, and also a fructose-biphosphate aldolase from the Calvin cycle pathway. The power of the RHM procedure was estimated as 47% when the causal quantitative trait loci generated 10% of the phenotypic variance (sample size = 451). Cassava DM genetics are complex and RHM may be useful for complex traits.

## Core ideas

- Regional heritability mapping (RHM) is effective for understanding the genetic architecture of complex traits in cassava.
- Prediction accuracies can reflect the impact of genomic segments on cassava dry matter (DM) content.
- Serine–threonine protein kinases (SnRKs) are candidates positionally associated with cassava DM.
- The prediction accuracy of SnRKs for cassava DM was 50% of the total accuracy from genome-wide single nucleotide polymorphisms.

**C**assava currently ranks as the sixth world staple crop consumed by more than 500 million people in Africa, Asia, and South America (El-Sharkawy, 2003). It was originally a perennial shrub but is cultivated now as an annual for its starchy root (El-Sharkawy, 2003). It is an outbreeding species and considered to be an amphidiploid or sequential allopolyploid (El-Sharkawy, 2003). The crop is clonally propagated by mature woody stem cuttings called stakes, which are 15 to 30 cm long and planted mostly inclined on ridged soils (Keating et al., 1988). Botanical seeds are used mainly in breeding programs with up to three seeds produced per pod (Iglesias et al., 1994, Iglesias and Hershey, 1991). Storage roots are generally harvested 7 to 24 mo after planting (El-Sharkawy, 2003). Dry matter is the major product from cassava roots apart from moisture and traces of water-soluble vitamins and pigments (Holleman and Aten, 1956; Barrios and Bressani, 1967; Lim, 1968). On average, cassava DM is made up of about 90% carbohydrates (mainly starch), 2% protein, 1% fat, 3% minerals and ash, and 4% fiber (Holleman and Aten, 1956; Barrios and Bressani, 1967; Lim, 1968). This starch deposit makes cassava attractive for the food industry and other industries that rely heavily on starch as their primary raw material (Lim, 1968). The value of cassava derives from a combination of fresh root yield and the percentage of DM that can be extracted from fresh roots, referred to as dry yield. Fresh cassava roots with a high DM content are also preferred by local farmers and processors (Kawano et al., 1987; Safo-Kantanka and Owusu-Nipah, 1992; Enidiok et al., 2008), who transform cassava roots into valuable staples consumed by many in developing countries. With 263 million metric tons produced in 2012 (Food and Agriculture Organization of the United Nations, 2013), cassava has become an indispensable staple in the world and improvement of cassava for high dry yield is needed. This improvement should also endeavor to increase micronutrient content, as it is much needed in the cassava-consuming regions of the world. Biofortification is a successful genetic improvement technique for increasing micronutrient content in staple crops (Meenakshi et al., 2010; Bouis et al., 2011) and represents a promising approach for solving the problem of micronutrient malnutrition around the world (Meenakshi et al., 2007, 2010; Pfeiffer and McClafferty, 2007).

The target of biofortification is to increase the content of essential micronutrients such as iron, zinc, and Vitamin A (Meenakshi et al., 2007, 2010; Pfeiffer and McClafferty, 2007), hence improving the health of millions of people who depend on these staples for daily nutrition. The biofortification process is facilitated by plant breeding (Meenakshi et al., 2010; Bouis et al., 2011). Since the early 2000s, the HarvestPlus initiative (Meenakshi et al., 2007; Pfeiffer and McClafferty, 2007) has been tasked with biofortification of staple crops including cassava, sweet potato [*Ipomoea batatas* (L.) Lam.], maize (*Zea mays* L.), rice (*Oryza sativa* L.), and wheat (*Triticum aestivum* L.). Biofortification of cassava is geared toward breeding varieties containing increased levels of Provitamin A, or β-carotene, a precursor for Vitamin A. The so-called ‘yellow cassava’ (Liu et al., 2010; HarvestPlus, 2009; Aniedu and Omodamiro, 2012; La Frano et al., 2013) is designed to address public health issues including child mortality, impaired vision and night blindness, reduced immunity to diseases, and other consequences of vitamin A deficiency (Liu et al., 2010; HarvestPlus, 2009).

Breeding for the required levels of Provitamin A necessitates the accumulation of β-carotene in cassava roots (Aniedu and Omodamiro, 2012; La Frano et al., 2013). Many breeding programs use yellow flesh color as a proxy for measuring the β-carotene levels in cassava despite the fact that yellowness is more of an indication of total carotenoids in the root (Chávez et al., 2005; Ssemakula et al., 2007; Akinwale et al., 2010). This protocol is used to visually preselect lines containing β-carotenoids prior to quantification of different carotenoid levels with high-performance liquid chromatography protocols (Kimura et al., 2007; Adewusi and Bradbury, 1993). Breeding for farmer-preferred biofortified cassava involves the development of high yielding clones with high DM and high β-carotene accumulation in a single clone or variety (Ceballos et al., 2004; Raji et al., 2007). Incorporating all these characteristics in a single variety of cassava makes for a challenging breeding task. Some studies have shown that there is a negative genetic correlation between DM and yellow root flesh color in cassava, making this breeding task even more challenging, since the target is toward full adoption of Provitamin A varieties by local farmers and processors (Akinwale et al., 2010; Vimala et al., 2009). It is therefore useful to understand the genetic control of DM content and β-carotene accumulation in cassava to facilitate the breeding of farmer-preferred varieties.

Regional heritability mapping is a relatively new procedure for identifying loci affecting quantitative traits (Nagamine et al., 2012; Riggio and Pong-Wong, 2014; Riggio et al., 2013; Shirali et al., 2015). Unlike single-marker genome-wide association analysis (GWAS) methods, which the lack power to detect rare genetic variants (Bodmer and Tomlinson, 2010; Gibson, 2012; Wood et al., 2014), RHM can capture both rare and common genetic variants, giving it more power to identify loci that cannot be detected by standard GWAS (Nagamine et al., 2012; Riggio and Pong-Wong, 2014; Riggio et al., 2013). Regional heritability mapping has been shown to detect both common and rare genetic variants implicated in disease traits in human genomics (Shirali et al., 2015; Uemoto et al., 2013; Zeng et al., 2016) and recently in tree genomics (Resende et al., 2017). Regional heritability mapping is a suitable method for capturing the effect of a genomic block or segment, since it can identify genomic segment–trait associations for regions spanning multiple loci (Nagamine et al., 2012; Riggio and Pong-Wong, 2014; Riggio et al., 2013; Caballero et al., 2015). A multimarker mapping approach like RHM may identify both common and rare variants involved in the expression of DM in white and yellow subpopulations of African cassava. To the best of our knowledge, this is the first attempt to use the RHM procedure in an annual crop.

The objectives of this study were:

1. To understand the genetic basis of DM in white- and yellow-rooted African cassava populations; and
2. To determine the power of the RHM procedure to detect genomic segments carrying quantitative trait loci via the “hide a causal single nucleotide polymorphism (SNP)” procedure.

## MATERIALS AND METHODS

### Cassava Phenotypic Data for Discovery

We used phenotypic data collected from the Genetic Gain (GG) population trials conducted by the cassava breeding program at the IITA, Ibadan, Nigeria for our analysis. The GG population (713 clones) is an elite population bred from the 1970s to 2007 by the cassava breeding program at the IITA (Maziya-Dixon et al., 2007; Okechukwu and Dixon, 2008; Ly et al., 2013). Most GG clones are of African origin with such good performance that they were advanced to multienvironment uniform yield trials. For this study, we used clonal evaluation trials of the GG population planted in an augmented design. The clonal evaluation trials use an unreplicated incomplete block design consisting of a layout of between 18 and 30 blocks with 22 accessions and two checks in each block. Accession plots were a single row (1 by 1m spacing) of five-plant stands without borders. All checks were included in the analysis. A few trials were replicated twice. These trials were conducted in three locations in Nigeria: Ibadan (7.40°N, 3.90°E), Mokwa (9.3°N, 5.0°E), and Ubiaja (6.66°N, 6.38°E) between 2013 and 2015. Three core agronomic traits were measured for these trials, including the fresh weight of harvested roots expressed in tons per ha (t ha^–1^) (fresh root yield, FYLD); the percentage of DM of storage roots, which measures root dry weight as the percentage of the root fresh weight; and pulp color a binary trait rated on a scale from 1 (white to light cream root flesh) to 2 (deep cream to yellow root flesh). The DM trait was measured via the oven method: 100 g of grated root sample (with thorough mixing of 10–15 randomly selected roots from a plot) were collected per accession and oven-dried. Dry matter content was then measured as residual weight after oven drying. We further divided the GG population (713 clones) into two subpopulations of white (451 clones) and yellow (262 clones) cassava with the pulp color trait where clones with a score of 1 for this trait were grouped into the white population and those with score 2 into the yellow population.

### Cassava Phenotypic Data Used for Validation

To validate the results from the RHM analysis, we used data from a population called the GS-C1, which consisted of the progeny of clones from the GG population described above. Phenotypes from the GS-C1 were obtained from clonal evaluation trials of 1651 clones split into trials at three locations: Ibadan, Mokwa, and Ikenne (6°52ʹN, 3°43ʹE). These trials were planted with an augmented design consisting of 20 to 30 blocks with 22 to 24 clones and two checks in each block. Plots were a single row of five-plant stands (1 by 1m spacing) without borders and without replication, and trials were planted during 2014 and 2015. Cassava trait measurements for this population were as described earlier, except that no strict distinction between yellow and white flesh color was used because the GS-C1 plants were mostly white and cream clones; thus we performed validation analysis with all clones.

### Cassava Genotype Data

DNA was extracted with the DNeasy Plant Mini Kits (Qiagen, Germantown, MD) from 713 clones from the 2013 Genetic Gain trial at IITA and was quantified with PicoGreen (Thermo Fisher Scientific, Waltham, MA). Genotyping-by-sequencing was used for genotyping (Elshire et al., 2011) these clones. Six 95-plex and one 75-plex *Ape*KI libraries were constructed and sequenced on an Illumina HiSeq (Illumina, San Diego, CA) , with one lane per library. Single nucleotide polymorphisms were called from the sequence data using the TASSEL pipeline Version 4.0 (Glaubitz et al., 2012) with an alignment to the *M. esculenta* Version 6 reference genome (Goodstein et al., 2012). The marker data were converted to a dosage format (0, 1, 2) and missing genotypic data were imputed with Beagle software (Ayres et al., 2011). The final dataset consisted of 177,201 SNPs scored in 713 clones. Members of the GS-C1 population used in the validation analysis were genotyped in 2014 as described above. Single nucleotide polymorphisms from both populations were called together with the TASSEL pipeline (Glaubitz et al., 2012) and missing genotypes also imputed with Beagle (Ayres et al., 2011), yielding the same number of SNPs as above.

### Data Analysis

#### Genome-wide RHM

Regional heritability mapping was performed using the following procedure (Nagamine et al., 2012; Riggio and Pong-Wong, 2014; Riggio et al., 2013):

- Chromosomes were divided into 100 SNP segments in sliding windows with 50 SNPs overlapping between adjacent windows.
- A multikernel univariate mixed model (Model 1; Eq. [1]–Eq. [7]) was used to partition the genomic additive variation caused by trait of interest into components of the target genomic segment and the whole-genome SNP markers as follows:
  $$y=x\text{β}+Zu_{1}+Zu_{2}+e;$$ 1
  $$u_{1}∼N\left(0,\;{\text{σ}}^{2}u_{1}Ku_{1}\right);$$ 2
  $$u_{2}∼N\left(0,\;{\text{σ}}_{u_{2}}^{2}Ku_{2}\right);$$ 3
  $$e∼N\left(0,\;{\text{σ}}_{e}^{2}I_{n\times n}\right);$$ 4
  $$\mathrm{var}(y)=V=Z\left({\text{σ}}^{2}u_{1}Ku_{1}\right)Z^{T}+Z\left({\text{σ}}^{2}u_{2}Ku_{2}\right)Z^{T}+\;{\text{σ}}_{e}^{2}I;$$ 5
  $$\overset{\wedge }{u_{1}}=\left({\text{σ}}^{2}{}_{u_{1}}K_{u_{1}}\right)Z^{T}V^{-1}\left(y-X\overset{\wedge }{\text{β}}\right);$$ 6
  $$\overset{\wedge }{u_{2}}=\left({\text{σ}}^{2}{}_{u_{2}}K_{u_{2}}\right)Z^{T}V^{-1}\left(y-X\overset{\wedge }{\text{β}}\right),$$ 7
  where *y* is a response variable (DM), *X* is a known incidence matrix for fixed effects β (including grand mean and a nested effect of trial within year within location), *Z* is a known incidence matrix for the clonal additive genomic effects *u_1_* for the target genomic segment and *u_2_* for the whole-genome SNPs. *K_u1_* and *K_u2_* are the genomic relationship matrices calculated from the SNPs with the procedure of VanRaden (2008) as:
  $$G=\frac{MM^{T}}{2\sum p\left(1-p\right)},$$ 8
  where *G* is the genomic relationship matrix; *M* is a centered marker matrix coded as -1,0,1; and *p* is the major allele frequency vector. Other components of the model include the genomic variance for the target genomic segment (σ_*u*_1__^2^ ) and the total genomic variance for the whole genome (σ_*u*_2__^2^); σ_*e*_^2^ is the genomic error variance and *e* indicates the residuals from the model. Model 1 was fitted using the R EMMREML package (Akdemir and Okeke, 2014). Note that the *K_u2_* genomic relationship matrix serves to control statistically for population structure effects, like the kinship matrix does in standard GWAS.
- Following model fitting in Step (b) above, the genomic heritability for each target genomic segment was computed as follows:
  $$h^{2}=\frac{{\overset{\prime }{o_{u_{1}}}}^{2}}{{\overset{\prime }{o_{u_{1}}}}^{2}+{\overset{\prime }{o_{u2}}}^{2}+{\overset{\prime }{o_{e}}}^{2}}.$$ 9
  where *h*^2^ is genomic heritability for a target genomic segment and the variance components are as described above.
- A likelihood ratio test was used to test the significance of target genomic segments with the alternative model as Model 1 and the null model as Model 1 without the target genomic kernel component i.e., *y* = *X* β + *Zu*_2_ + *e* . This model was also fitted with the EMMREML package (Akdemir and Okeke, 2014). *P*-values were obtained with the *pchisq* function in R (R Development Core Team, 2016).
- Local false discovery rate (LFDR) was estimated with the R qvalue package (Storey and Tibshirani, 2003; Storey et al., 2015).
- Genomic segment LFDRs were then plotted across the genome in a Manhattan plot with a cutoff of 0.05 being used to assess significance.

We performed the RHM procedure separately for the white and yellow cassava subpopulations of GG. No defined population structure was found on in the GG population in a previous GWAS study (Wolfe et al., 2016). Therefore, the genomic relationship matrix from the whole-genome SNPs in the RHM was sufficient to account for structure in this analysis (in fact, we refer to this more as background effect).

### Candidate Gene Analysis

We identified candidate genes from the significant hits of the RHM analysis based on annotations for the Version 6 *M. esculenta* genome on phytozome (Goodstein et al., 2012). We used plant physiology information to narrow down the list of genes associated with carbohydrate biosynthesis, including genes that are functional in starch and sugar biosynthesis, cell wall loosening and degradation, and root sink and plant growth pathways. We performed validation tests on candidates selected on the basis of their prediction accuracies in the GS-C1 population as described below.

### Validation Models and Procedures

We conducted validation analyses for the significant hits from the RHM analysis and for the RHM procedure itself. Validation here was geared toward understanding the prediction accuracies obtained from genes and gene families on significant RHM segments. Validation proceeded as described below.

#### Validation with SnRK Genes (a Candidate Gene Family)

To obtain genotypic data for this analysis, we searched the Phytozome *M. esculenta* Version 6.1 web portal (Goodstein et al., 2012) using the keyword ‘serine threonine kinases’ to recover all instances of this in the cassava genome, resulting in 2408 hits. We filtered the resulting list to remove all hits not containing gene ontology or eukaryotic orthologous group function definitions for the keyword ‘serine threonine kinase’. We then manually added genes containing known serine threonine kinases that did not contain a function definition, such as the *sucrose nonfermenting 1* gene (a list of these genes is provided in the Supplemental Table S1). We extracted all markers within 2.5 kb of the start and end of each gene model with the Bedtools intersect function (Quinlan and Hall, 2010), resulting in 7203 unique SNPs. We refer to these SNPs as candidate SNPs below. For validation of these candidate SNPs on the GS-C1 data, we fitted the following model (Model 2, Eq. [10]–Eq. [16]):

y=Xβ+Zs+Zg+e; 10

$$s∼N\left(0,{\text{σ}}_{s}^{2}K_{s}\right);$$ 11

$$g∼N\left(0,{\text{σ}}_{g}^{2}K_{g}\right);$$ 12

$$e∼N\left(0,{\text{σ}}_{e}^{2}I_{n\times n}\right);$$ 13

$$\mathrm{var}(y)=V=Z\left({\text{σ}}_{s}^{2}K_{s}\right)Z^{T}+Z\left({\text{σ}}_{g}^{2}K_{g}\right)Z^{T}+\;{\text{σ}}_{e}^{2}I;$$ 14

$$\overset{\wedge }{s}=\left({\text{σ}}^{2}{}_{s}K_{s}\right)Z^{T}V^{-1}\left(y-X\overset{\wedge }{\text{β}}\right);$$ 15

$$\overset{\wedge }{g}=\left({\text{σ}}^{2}{}_{g}K_{s}\right)Z^{T}V^{-1}\left(y-X\overset{\wedge }{\text{β}}\right);$$ 16

where *y* is a vector of the raw phenotypic values for DM, *X* is the known incidence matrix for fixed effects β (including grand mean and a nested effect of trial within year within location), *Z* is known incidence matrix for clonal additive candidate genomic effects *s* and whole-genomic effects *g*. For *K_s_* and *K_g_*, we used the candidate SNPs and the remaining SNPs from the whole genome excluding the candidate SNPs, respectively, to generate genomic relationship matrices for the 1651 clones of the GS-C1 population as above. A third kinship matrix, *K*_rand_, was generated as a control from 7203 SNPs anchored to 2000 randomly selected genes from the cassava genome and used in Model 2 in place of *K*_s_, whereas we calculated *K_g_* by using SNPs from the whole genome excluding those in *K*_rand_. Other components of the model include the SnRK candidates’ genetic variance (σ*_s_*^2^) and the genetic variance from other parts of the genome (σ*_g_*^2^), σ*_e_*^2^ is the error variance, and *e* represents the residuals from the model. Model 2 was fitted with the EMMREML package. To assess prediction accuracies, we fitted another model as follows (Model 3, Eq. [17]–Eq. [21]):

y=Xβ+Zu+e; 17

$$u∼N\left(0,{\text{σ}}_{u}^{2}I\right);$$ 18

$$e∼N\left(0,{\text{σ}}_{e}^{2}I_{n\times n}\right);$$ 19

$$\mathrm{var}\left(y\right)=V=Z\left({\text{σ}}_{u}^{2}K\right)Z^{T}+{\text{σ}}_{e}^{2}I;$$ 20

$$\overset{\wedge }{u}=\left({\text{σ}}^{2}{}_{u}K\right)Z^{T}V^{-1}\left(u-X\overset{\wedge }{\text{β}}\right),$$ 21

where most components of Model 3 remain same as those in Model 2, apart from the genetic effect *u* having an identity matrix *I* as its covariance matrix, signifying that the 1651 GS-C1 validation clones were unrelated. Model 3 was also fitted with the R EMMREML package. Model 2 was fitted using a fivefold a cross-validation scheme with 10 repeats, and prediction accuracies were obtained for this cross-validation scheme by a correlation of *s*ˆ of each clone from Model 2 to its *u*ˆ value from Model 3.

#### Validation with 53 Candidate Genes Extracted from Plant Physiology Literature and 53 Randomly Selected Genes from the Significant RHM Regions

We performed a second procedure to validate the 53 candidate genes identified in significant hit regions in the RHM analysis based on plant physiology literature (Table 1). Using the cassava genome’s unique gene identifiers from Phytozome (Goodstein et al., 2012), we extracted all markers within 2.5 kb flanking the start and end of each gene as before, resulting in 400 unique SNPs. We refer to these SNPs as ‘likely candidate SNPs’. We also picked 53 single-copy genes at random from within the significant RHM regions and anchored them to 395 SNPs as controls for the likely candidate SNPs. We term these the ‘unlikely candidate SNPs’. To validate these, we also fitted the Genomic Best Linear Unbiased Prediction Model 2 with the following modifications: (i) for *K_s_*, we used *K_53_*_,_ which was a genomic relationship matrix calculated from the 400 likely candidate SNPs for the 1651 clones of the GS-C1 population (as above); (ii) we calculated *K_g_* with SNPs from the whole genome excluding the likely candidate SNPs; (iii) *K_rand_* was also calculated as above (as a control) from 402 SNPs anchored to 53 randomly selected genes from the cassava genome (with 7.5 kb flanking the start and end of these genes); (iv) *K_unlikely_* was calculated from the 395 unlikely candidate SNPs. These were also used in place of *K_s_* in Model 2 with their appropriate *K_g_* being calculated as other SNPs in the genome excluding those in *K_rand_* and *K_unlikely_*. Other components of the model were as described for Model 2 and prediction accuracies were obtained in the same way. To assess the prediction accuracy of the whole-genome SNPs, we also fitted a model analogous to Model 3 with the covariance of *u* coming from a genomic relationship matrix with whole-genome SNPs. We term this the predictive accuracy of the whole-genome SNPs.

**Table 1:** Candidate cassava genes and gene families associated with significant regional heritability mapping (RHM) regions.

RHM candidates for white DM											
Chr.†	Segment tag SNP_ID	Segment tag SNP	Segment span	LFDR	Target segment variance	Genomic segment variance	Candidate genes	Phytozome ID	Start	End	Homologs
		bp							——bp——		
1	S1_3416180	3416180	3,064,700–3,416,180	0.047227533	0.66	0.84	*BAK1-interacting receptor-like kinase 1*	*Manes.01G018900*	3,095,915	3,098,209	*AT5G48380.1*
							*BAK1-interacting receptor-like kinase 1*	*Manes.01G019000*	3,132,412	3,134,588	*AT5G48380.1*
1	S1_19936380	19936380	1,964,6964–19936380	0.030168971	0.58	0.82	*UDP-D-glucuronate 4-epimerase 2*	*Manes.01G074000*	19,949,560	19,951,824	*AT1G02000.1*
							*DHHC-type zinc finger family protein*	*Manes.01G073100*	19,818,085	19,825,785	*AT3G60800.1*
							*Bifunctional inhibitor trypsin-alpha amylase inhibitor*	*Manes.01G074600*	20,036,878	20,037,858	*AT1G62790.2*
							*Plant invertase/pectin methylesterase inhibitor superfamily protein*	*Manes.01G074800*	20,042,476	20,043,982	*AT4G25260.1*
							*Pectinesterase*	*Manes.01G075600*	20,158,111	20,159,120	*AT5G07420.1*
							*beta vacuolar processing enzyme*	*Manes.01G075700*	20,173,648	20,176,768	*AT1G62710.1*
							*Mini zinc finger 2*	*Manes.01G090800*	21,547,715	21,548,005	*AT3G28917.1*
							*Galactosyltransferase1*	*Manes.01G091600*	21,599,298	21,603,804	*AT1G26810.1*
							*Starch synthase 2 (ADP-glucose type)*	*Manes.01G091700*	21,623,316	21,629,007	*AT3G01180.1*
							*Cycling DOF factor 2*	*Manes.01G092100*	21,647,142	21,650,438	*AT5G39660.1*
4	S4_537966	537966	510,971–732,069	0.003820074	0.67	0.84	*SnRK1 RIO*	*Manes.04G004800*	535,401	538,252	*AT1G08290.1*
4	S4_859155	859155	623,461–859,155	0.00113334	0.62	0.82	*Alkaline/neutral invertase*	*Manes.04G006900*	778,931	784,740	*AT5G22510.1*
							*Cellulose synthase-like A02*	*Manes.04G009400*	1,064,431	1,069,211	*AT5G22740.1*
							*C2H2-like zinc finger protein*	*Manes.04G008800*	962,985	963,932	*AT4G35280.1*
							*(SnRK1)*	*Manes.04G006600*	757,312	760,778	*AT3G44610.1*
5	S5_7279374	7279374	7,061,162–7,279,374	0.03310678	0.69	0.85	*Trehalose-phosphatase/synthase 9*	*Manes.05G087900*	6,905,372	6,911,159	*AT1G23870.1*
							*SnRK1 With No Lysine-related*	*Manes.05G089000*	7,284,514	7,286,444	*AT1G60060.1*
							*SnRK1*	*Manes.05G090100*	7,365,593	7,370,118	*AT1G24030.2*
							*NAD(P)-linked oxidoreductase superfamily protein*	*Manes.05G092400*	7,630,814	7,632,642	*AT1G59960.1*
							*Galactosyltransferase family protein*	*Manes.05G095000*	7,849,628	7,853,700	*AT5G62620.1*
							*bHLH transcription factor*	*Manes.05G094700*	7,824,312	7,824,497	*LOC_Os07g09590.1*
							*Galactose oxidase*	*Manes.05G096300*	7,992,476	7,993,801	*Cre06.g306000.t1.1*
							*Alpha-amylase-like 3*	*Manes.05G097100*	8,094,930	8,103,684	*AT1G69830.1*
							*sucrose transport protein (SUC3)*	*Manes.05G099000*	8,344,933	8,345,349	*LOC_Os02g36700.1*
							*UDP-galactose/UDP-glucose transporter*	*Manes.05G101600*	8,633,269	8,636,565	*Potri.016G139100.1*
							*Zinc finger, C3HC4 type domain containing protein*	*Manes.05G102000*	8,696,530	8,696,856	*LOC_Os03g20870.1*
10	S10_15606779	15606779	1,502,4128–15,606,779	0.041702151	0.15	0.64	*SnRK1*	*Manes.10G079500*	14,844,954	14,851,620	*AT1G49730.1*
							*bHLH DNA-binding superfamily protein*	*Manes.10G080300*	15,279,304	15,281,955	*AT1G49770.1*
							*Salt tolerance zinc finger*	*Manes.10G080000*	15,071,612	15,072,680	*AT1G27730.1*
							*Protein with RNI-like/FBD-like domains*	*Manes.10G080200*	15,240,978	15,241,397	*AT5G56410.1*
							*Regulator of Vps4 activity in the MVB pathway protein*	*Manes.10G080400*	5,346,375	15,347,741	*AT2G14830.2*
17	S17_5368263	5368263	5,152,794–5,368,263	0.005975712	0.17	0.64	*Xyloglucan endotransglucosylase/hydrolase 30*	*Manes.17G015100*	5,198,786	5,201,174	*AT1G32170.1*
17	S17_5532660		5,285,956–5,532,660	0.005529599	0.27	0.67	*Pectin lyase-like superfamily protein*	*Manes.17G015200*	5,264,305	5,276,255	*AT3G07840.1*
							*bHLH DNA-binding family protein*	*Manes.17G016000*	5,657,214	5,660,207	*AT1G32640.1*
17	S17_16084946	16084946	15,509,115–16,084,946	0.046144077	0.14	0.62	*CBL-interacting protein kinase 23 (SnRK3)*	*Manes.17G073900*	21,282,260	21,287,846	*AT1G30270.1*
							*Inositol transporter 2*	*Manes.17G073000*	21,211,865	21,215,459	*AT1G30220.1*
							*Transducin/WD40 repeat-like superfamily protein*	*Manes.17G073300*	21,251,484	21,254,389	*AT1G65030.1*
18	S18_4988720	4988720	4,586,953–4,988,720	0.037726745	0.69	0.85	*Sucrose transporter 4*	*Manes.18G054200*	4,548,075	4,559,586	*AT1G09960.1*
							*Nucleotide-diphospho-sugar transferases superfamily protein*	*Manes.18G054400*	4,572,586	4,576,353	*AT1G27600.1*
							*beta HLH protein 93*	*Manes.18G055000*	4,655,203	4,657,015	*AT5G65640.1*
							*CBL-interacting protein kinase 8 (SnRK3)*	*Manes.18G055300*	4,677,895,	4,683,026	*AT4G24400.1*
							*UDP-glycosyltransferase superfamily protein*	*Manes.18G056200*	4,793,379	4,795,015	*AT5G49690.1*
							*GATA-type zinc finger protein with TIFY domain*	*Manes.18G056300*	4,820,431	4,826,234	*AT4G24470.3*
**RHM candidates for yellow DM**											
1	S1_22566390	22566390	21,910,139–22,566,390	0.04682523	0.72	0.87	*Galacturonosyltransferase 13*	*Manes.01G098400*	22,245,408	22,256,933	*AT3G01040.1*
							*Plant calmodulin-binding protein-related*	*Manes.01G099000*	22,282,570	22,286,073	*AT5G39380.1*
							*UDP-glucosyl transferase 76E11*	*Manes.01G100300*	22,370,079	22,371,643	*AT3G46670.1*
							*SNF1 kinase (SnRK1)*	*Manes.01G100900*	22,409,977	22,417,397	*AT3G01090.2*
							*Vesicle transport v-SNARE family protein*	*Manes.01G101200*	22,421,446	22,425,558	*AT5G39510.1*
							*Brassinosteroid-6-oxidase 2*	*Manes.01G102800*	22,514,594	22,518,856	*AT3G30180.1*
9†	S9_13325846	13325846	12,810,619–13,747,638		0.26	0.66	*Phosphofructokinase 2*	*Manes.09G077800*	13,223,145	13,226,580	*AT5G47810.1*

† Strong but nonsignificant signal.

‡ Chr., chromosome, FDR, false discovery rate; DM, dry matter; SNP, single nucleotide polymorphism; SnRK, serine–threonine protein kinase; bHLH, basic helix-loop-helix; DoF, DNA-binding one zinc finger; LFDR, local false discovery rate; UDP, uridine diphosphate; ADP, adenosine diphosphate; RIO, right open reading frame; NAD(P), nicotinamide adenine dinucleotide phosphate; MVB, multivesicular body; CBL, calcineurin B-like proteins; GATA, erythroid transcription factor; HLH, helix–loop–helix; SNARE, soluble N-ethylmaleimide-sensitive factor (NSF) attachment protein receptor.

#### Validation With All Genes within 1 Mb of the Significant RHM List and an a priori List of Starch Genes in Cassava

We performed another validation procedure to provide a validation for all the genes identified in the significant hit regions in the RHM analysis, including those shown in Table 1 and those not shown because they were not selected on the basis of the information from the literature. By using the cassava genome’s unique gene identifiers from Phytozome (Goodstein et al., 2012), we extracted all SNPs within a 1-Mb region centered on each of these candidates with Bedtools (http://bedtools.readthedocs.io/en/latest/, accessed 20 Nov. 2017), resulting in 2297 SNPs from 650 unique genes. We refer to these SNPs as the RHM-region SNPs. In addition, we extracted the SNPs anchored to 123 unique genes in the cassava starch pathway compiled by Saithong et al. (2013), resulting in 419 SNPs. We refer to these SNPs as cassava starch SNPs. To validate these SNPs, we fitted Model 2 with genomic relationship matrices calculated as above from the RHM-region and cassava starch SNPs, in place of *K_s_*, with their appropriate *K_g_* calculated from remaining SNPs. We also picked 650 single-copy genes at random, excluding the significant RHM regions, and anchored them to approximately 2300 SNPs as controls for the RHM-region and cassava starch SNPs. We refer to these as Random-650 SNPs. We calculated *K_random-650_* from these SNPs and an appropriate *K_g_*. These kernels were also fitted in Model 2 as *K_s_* and *K_g_*, respectively. In addition to the prediction accuracies from these candidates, we validated genes in the RHM-region set by searching for them in two a priori lists compiled by Saithong et al. (2013), including one for the cassava starch pathway and another for the Calvin cycle pathway. The RHM-region genes that made this list were considered to be validated.

#### Assessing the RHM’s Power via the “Hide a Causal SNP” Procedure

To validate the RHM procedure, we performed an analysis similar to the classical “hide a causal SNP” approach as follows:

- Chromosomes were divided into 100 SNP segments in sliding windows with 50 SNPs overlapping between adjacent segments.
- Five adjacent segments were randomly selected on each chromosome.
- On the third segment, effects were added to a random SNP to inflate the phenotypic variance of the DM trait by 10%.
- Genomic relationship matrices were made for these segments but for Segment 3, the random pseudocausal SNP was excluded during calculation of the genomic relationship matrix.
- Subsequently, Steps (b) to (d) of the RHM procedure above were performed, resulting in *P*-values for these five adjacent segments. Steps (a) to (e) were repeated twelve times, resulting in 216 tests.
- We then calculated the *P*-value from the RHM analysis on our data that corresponded to the LFDR threshold of 0.05 and used this as the significance threshold.
- The power of the RHM analysis was then calculated as the number of times any of the five segment *P*-values were significant, given the significance threshold from Step (f) above.
- To make a decision on the bounds set for extracting adjacent candidate genes from the *M. esculenta* genome for a significant segment in the RHM analysis, the number of times either the first or fifth segment’s *P*-values were significant, conditional on the third segment having a higher *P*-value, was also calculated. This reflected how far away adjacent segments captured causal variants.

## RESULTS

### Regional Heritability Mapping for DM in White and Yellow Cassava Populations

**Fig. 1:**
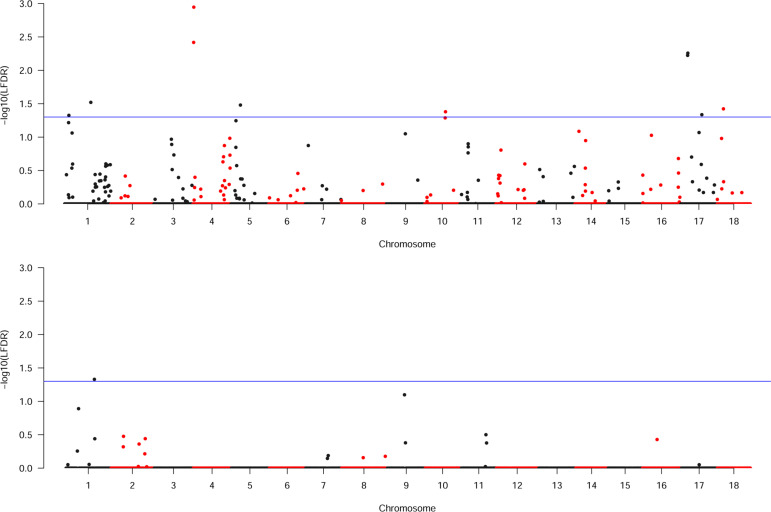
Manhattan plots showing dry matter content and genomic segment associations in cassava. The upper and lower figures show regional heritability mapping (RHM) discovery associations for white and yellow cassava populations, respectively.

The genomic heritabilities for DM in white and yellow cassava, based on whole-genome SNPs, were 0.57 and 0.48, respectively. These heritabilities are somewhat higher than those found by Ly et al. (2013), presumably because they worked with more locations and years, and thus experienced higher genotype × environment interactions. We observed different genetic control patterns for DM in the white and yellow cassava subpopulations, as shown by the Manhattan plots from the RHM analysis (Fig. 1).

Significant genomic segments for the white cassava DM were observed on chromosomes 1, 4, 5, 10, 17, and 18; for the yellow cassava, a significant segment was only observed on chromosome 1 (Fig. 1). Because of the difference between the sample sizes of both subpopulations, it is unclear if the DM genetic control patterns between these subpopulations were different. A nonsignificant but strong signal was also observed on chromosome 9 of both cassava subpopulations.

### Candidate Gene Analysis

**Fig. 2:**
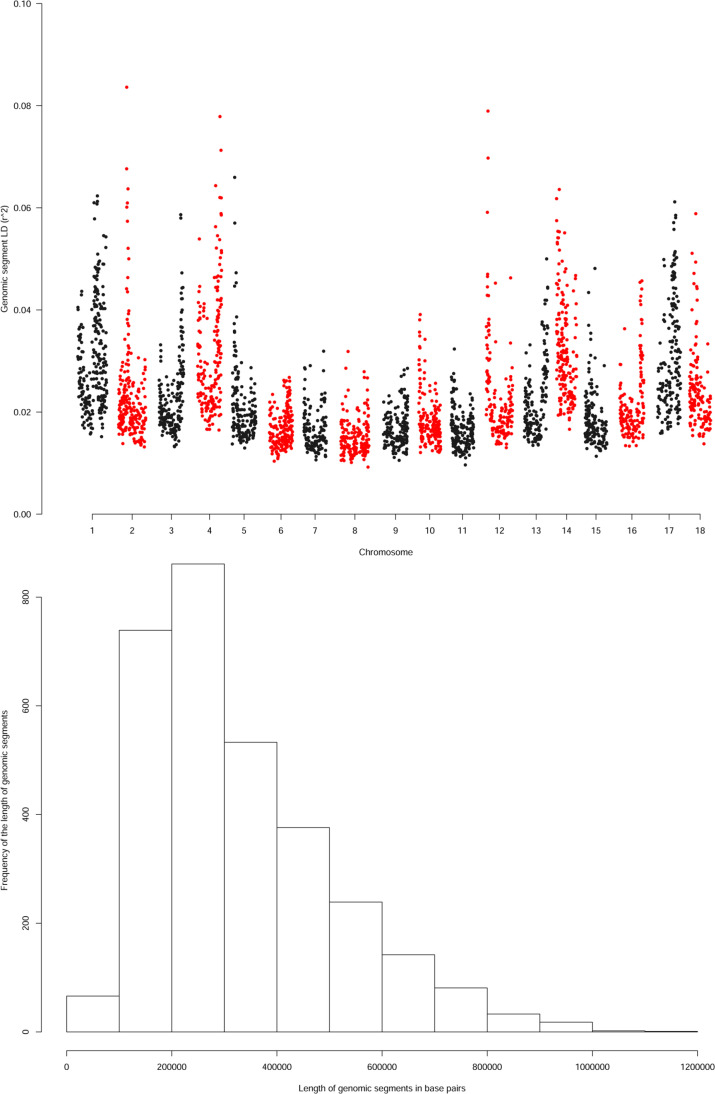
(A) Genome-wide linkage disequilibrium between segments in the regional heritability mapping (RHM) analysis of cassava. Linkage disequilibrium is measured as the mean correlation between all pairs of single nucleotide polymorphisms (SNPs) where one SNP is on one segment and the other is on the adjacent segment. (B) Histogram of the size of genomic segments in the RHM analysis. The size of the window is the physical distance in bp between the first and the last of the 100 SNPs in the window.

By using information from the estimates of the mean linkage disequilibrium between genomic segments per chromosome (Fig. 2A), the distribution of the length of genomic segments in our analysis (Fig. 2B), and information on the number of times adjacent segments captured causal variations in the simulation analysis, we set the bound for the region where candidate genes were sought to 1.0 Mb (500 kb flanking each hit), representing from two to three genomic segments adjacent to the top hit genomic segment.

### Candidates for the White and Yellow Cassava Subpopulations

For the top RHM hits in both cassava gene pools, we identified possible candidate genes and transcriptional regulators adjacent to these hits based on their involvement in the carbohydrate biosynthesis pathway, including members of the SnRKs family, members of the UDP-glycosyltransferase family (including starch and sucrose synthases), and UDP-sugar transporters; specific plant transcriptional factors including members of the β helix-loop-helix (bHLH) family and mini-zinc fingers; and other genes involved in cell wall processes, root storage, and development including pectinases and β-vacuolar processing enzymes. We show a list of these genes in Table 1. An additional candidate gene, *phosphofructokinase*, was associated with the nonsignificant peak on chromosome 9, which was more pronounced in the yellow cassava germplasm.

### Validation Results for SnRKs

The predictive accuracy of the whole-genome SNPs was 0.54 (SD of the cross-validation repeat cycle = 0.03). With the set of candidate SnRK SNPs, the prediction accuracies from the cross-validation with Model 2 were 0.26 (SD of the cross-validation repeat cycle = 0.04) and 0.12 (SD of the cross-validation repeat cycle = 0.06) for the candidate and random SNPs, respectively. The predictive ability of the genome-wide SnRK candidates (7203 SNPs) had ~50% of the total prediction accuracy from our set of genome-wide SNPs (177,201) for the GS-C1 population.

### Validation With 53 Likely Candidate Genes Extracted from Plant Physiology Literature and 53 Unlikely Candidate Genes from the Significant RHM Regions

With the likely candidate SNPs from the genes identified for all the top hitting genomic segments genome-wide (shown in Table 1), prediction accuracies from the cross-validation with a modified Model 2 were 0.17 (SD of the cross-validation repeat cycle = 0.03), those for the 53 unlikely genes randomly selected from the top hitting genomic segments genome-wide were 0.14 (SD of the cross-validation repeat cycle = 0.02) and those for the SNPs from 53 random genes from the cassava genome were 0.06 (SD of the cross-validation repeat cycle = 0.08).

### Validation With all Genes within 1 Mb of the Significant RHM List and an a priori List of Starch Genes in Cassava

Using the RHM-region, cassava starch, and Random-650 SNPs, the prediction accuracies from the cross-validation with a modified Model 2 were 0.17 (SD = 0.04), 0.18 (SD = 0.03), and 0.03 (SD = 0.01), respectively. Based on two a priori lists compiled by Saithong et al. (2013), including one for the cassava starch pathway and another for the Calvin cycle pathway, we found three RHM-region genes on the cassava starch pathway list, including an acid invertase (*Manes.01G076500*), a glucose-6-phosphate isomerase (*Manes.18G060600*), and a neutral or alkaline invertase (*Manes.04G006900*). However, in the Calvin cycle pathway list, we found one RHM-region gene, namely fructose-biphosphate aldolase (*Manes.04G007900*). These genes are known to play key roles in starch biosynthesis and storage (Junker, 2004; Ap Rees, 1992; Appeldoorn et al., 1997; Renz et al., 1993). To assess if these genes were significantly enriched in the RHM regions, we performed a simple calculation by multiplying the 650 genes in the RHM region by the 123 genes in the cassava starch pathway (Saithong et al., 2013) and divided them by the total number of genes in the cassava genome (33,030). The result was 2.4, which is the expectation of a Poisson process of obtaining the genes in the cassava starch pathway. However, we calculated the probability of drawing three cassava starch pathway genes from the genome at random, resulting in *P* = 0.22, indicating no significant enrichment.

### Assessing the RHM’s Power via the “Hide a Causal SNP” procedure

We calculated the statistical power of the RHM procedure to detect simulated causal effects from 216 analyses as the number of times any of the five segments’ *P*-values were significant. The *P*-value from the RHM analysis on our data that corresponded to the LFDR threshold of 0.05 was 0.00024, which became our significance threshold for this analysis. We found that 102 tests were significant out of a total of 216, representing 47% statistical power to detect the simulated causal region. To set the bounds for how far in the genome to cover when extracting candidate genes from a significant RHM segment, we also calculated the number of times the *P*-values from the first or fifth genomic segments were significant, conditional on the third segment’s *P*-value being higher. With a total of 216 analyses, 27 cases had significant *P*-values on Segment 3 and 15 cases had significant *P*-values from Segments 1 or 5 when the *P*-values from Segment 3 were higher. This represents 15% coverage further away from the causal segment. With this information, we chose an adjacent span of 500,000 kb pairs flanking a significant RHM segment as the bounds for extracting adjacent candidate genes. A summary of the prediction accuracies from validated candidates are shown in Table 2

**Table 2:** Summary of validation results for regional heritability mapping (RHM) significant candidates. Prediction accuracies from selected candidate genes or genomic segments were used to validate the significance of the RHM hits are given with the SD of cross-validation repeat cycles in parentheses.

Candidate†	Genomic segment	Prediction accuracy
SnRKs	7203	0.26 (0.04)
Random control for SnRKs	7203	0.12 (0.06)
53 Likely candidates	400	0.17 (0.03)
53 Unlikely candidates	395	0.14 (0.02)
Random control for 53 candidates	402	0.06 (0.08)
RHM-region genes	2297	0.17 (0.04)
Cassava starch genes	419	0.18 (0.03)
Random-650	2300	0.03 (0.01)
Whole genome SNPs	177,201	0.54 (0.03)

† SnRK, serine–threonine protein kinase; SNP, single nucleotide polymorphism.

## DISCUSSION

The RHM results in the high DM and white cassava populations clearly demonstrate the polygenic nature of the DM trait. Dry matter is composed of carbohydrates (mostly starch), cell wall components, and fiber, as well as other nonstarchy polysaccharides. Thus it is not surprising that this trait is complex and controlled by many genes. In addition, the RHM procedure in this study showed a 47% power for detecting association with a sample size of less than 500, given the polygenic nature of this trait.

### Serine–Threonine Protein Kinases may be Involved in the Regulation of Cassava Carbohydrate Biosynthesis

**Fig. 3:**
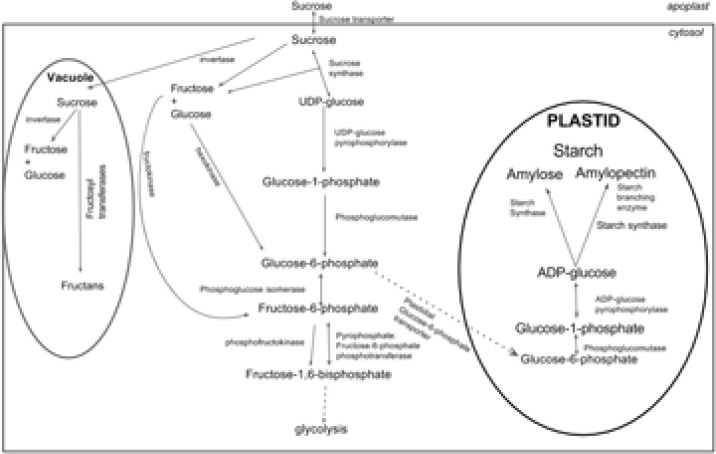
Sucrose and starch metabolism in a heterotrophic plant cell of the cassava tuber. Key enzymes including sucrose transporter, invertase, phospho-glucose isomerase, and phosphofructokinase were within 500 kb of significant regional heritability mapping (RHM) segments.

The SnRK gene family in plants is homologous to the sucrose nonfermenting 1 protein kinase family in yeast and the adenosine monophosphate-activated protein kinase gene family in mammals. Its members have gained recognition as critical elements in transcriptional, metabolic, and developmental regulation in plants (Halford et al., 2003; Halford and Hardie, 1998; Polge and Thomas, 2007; Xue-Fei et al., 2012; Crozet et al., 2014; Jossier et al., 2009). The most studied member of this family is SnRK1 (Halford and Hardie, 1998; Polge and Thomas, 2007). Serine–threonine protein kinases play a vital role as global regulators of C metabolism and mediate cross-talk between metabolic and other plant signaling pathways (Halford and Hardie, 1998; Polge and Thomas, 2007; Xue-Fei et al., 2012). SnRK1 was shown to play a key role in seed filling and maturation and in embryo development in pea (*Pisum sativum* L.) (Radchuk et al., 2006, 2010). In potato (*Solanum tuberosum* L.) and wheat, SnRK1 phosphorylates and inactivates key enzymes in the sugar and starch biosynthesis pathway, affecting sucrose synthase, trehalose phosphate synthase, and α-amylase (Purcell et al., 1998; Laurie et al., 2003). In potato, it stimulates the redox activation of adenosine 5’-diphosphate (ADP)-glucose pyrophosphorylase in response to high sucrose levels (Geigenberger, 2003; Tiessen et al., 2003). Antisense expression of SnRK1 resulted in a reduction in the expression of sucrose synthase in potato tubers (Purcell et al., 1998) and α-amylase in cultured wheat embryos (Laurie et al., 2003). However, the overexpression of SnRK1 in potatoes resulted in a significant increase in starch accumulation in tubers and a decrease in glucose levels resulting from a dramatic increase in the activity and expression levels of sucrose synthase and ADP-glucose pyrophosphorylase (McKibbin et al., 2006). SnRK1 is activated by high cellular sucrose or low glucose, or a dark period (Rolland et al., 2002). The model of sugar and starch biosynthesis in potato from McKibbin et al. (2006) showed SnRK1 to be at the heart of these processes. By using RHM analysis in the white cassava population, we identified significant genomic segments containing some of the proteins or enzymes in the model given in this illustration (McKibbin et al., 2006) including SnRKs, UDP-glycosyltransferases and UDP-sugar transporters, an ADP-type starch synthase 2, and a neutral invertase. Glycosyltransferases are a family of enzymes involved in carbohydrate biosynthesis of which sucrose and starch synthases are members (Momma and Fujimoto, 2012). With the RHM procedure and candidate gene analysis, several of these known carbohydrate biosynthesis enzymes (Table 1, Fig. 3) were putatively associated with the cassava DM trait.

### Other Possible Candidates that are Involved in Sugar and Starch Biosynthesis in Cassava

Other proteins located within significant genomic segments that are also involved in the carbohydrate biosynthesis pathway include invertase inhibitors, which have been shown to form complexes with SnRKs and lead to reduced accumulation of reducing sugars and increased accumulation of starch in potatoes (Lin et al., 2015), and BAK1, a brassinosteroid insensitive 1-associated receptor-like kinase and a member of the somatic embryogenesis receptor-like kinase (SERK) subfamily involved in regulation of root development (Du et al., 2012). BAK1/SERK1 positively controls starch granule accumulation in *Arabidopsis thaliana* (L.) Heynh. root tips (Du et al., 2012). With a transgenic sweet potato overexpressing a DNA-binding one zinc finger protein encoded by a *SRF1* gene [a member of the mini-zinc finger family of plant-specific transcription factors (Takatsuji, 1998, 1999)], Tanaka et al. (2009) showed that transgenic roots had significantly higher DM content in storage roots, increased starch content per unit of storage root fresh weight, and a drastic decrease in glucose and fructose levels. *SRF1* was shown to modulate carbohydrate metabolism in sweet potato storage roots via negative regulation of vacuolar invertase (Tanaka et al., 2009). Several enzymes, including pectinases, pectin esterases, cellulase synthase, and galacturonosyltransferases, found in the significant RHM regions in white and yellow cassava may be involved in plant cell wall loosening and degradation which may be linked to C partitioning in cassava. In fact galacturonosyltransferase, a member of the CAZy (Cantarel et al., 2009) GT8 family of glycosyltransferases, is involved in pectin and hemicellulose biosynthesis (Cantarel et al., 2009; Atmodjo et al., 2011; de Godoy et al., 2013). Galacturonosyltransferase-silenced tomato (*Solanum lycopersicum* L.) fruits showed altered pectin composition and decreased starch accumulation (de Godoy et al., 2013). Cassava galacturonosyltransferases may interfere with C metabolism, partitioning, and allocation, as seen in tomato (de Godoy et al., 2013). In their expression profile study using samples from different stages of cassava root development, Yang et al. (2011) found a significant upregulation of the enzymes involved in plant cell wall loosening and degradation. The bHLH family of transcription factors is a large family in plants involved in flavonoids, the carotenoid pathway, and anthocyanin pigmentation of tuber skin and flesh (from yellow to white and purple) in potato (De Jong et al., 2004; Zhang et al., 2009; Tai et al., 2013) and may interact with sucrose transporter to perform this function (Krügel et al., 2012). Phytochrome-interacting factors form a subfamily of bHLH transcription factors and PIF1 (a member of this subfamily) has been shown to directly regulate the expression of phytoene synthase (Toledo-Ortiz et al., 2010), a major driver of carotenoid production in plants and the first and main rate-determining enzyme of the carotenoid pathway (Toledo-Ortiz et al., 2010; Maass et al., 2009). It is not clear how bHLH may link with sugar biosynthesis and transport or play a role in starch accumulation in yellow cassava clones, but this may translate to the frequently observed negative correlation between DM and yellow root flesh color in African cassava (Esuma et al., 2016; Akinwale et al., 2010). Interestingly, cassava breeders in Colombia have not found any negative correlation between carotenoids and DM in their germplasm, and, in fact, have made gains in both traits by using a rapid cycling recurrent selection scheme (Ceballos et al., 2013).

### Some Experimental Studies that Reflect Possible Roles of Candidate Genes in the Cassava Tuber

By using the RHM analysis, we identified (Fig. 3) a number of cassava genes in the heterotrophic plant cell starch or sucrose metabolism pathway (Junker, 2004). We describe a few steps in this pathway, concentrating mostly on where we have identified candidate genes (candidate genes are in parentheses henceforth with phytozome gene identifiers). After sucrose is imported into the cytosol by a sucrose transporter (Manes.05G099000, Manes.18G054200), it is converted into hexose sugars via two paths involving the enzymes sucrose synthase (shown in the center of Fig. 3) and invertase (shown to the left in Fig. 3) (Manes.04G006900, Manes.01G076500) (Junker, 2004; Ap Rees, 1992; Appeldoorn et al., 1997; Renz et al., 1993).

Sucrose transport is much more pronounced in the sink tissues that switch to storage mode (Weschke et al., 2000, 2003). A transgenic study using *sucrose transporter 4-RNAi* mutant potato plants showed an increase in tuber yield and starch accumulation, and also induced early tuberization (Chincinska et al., 2008). It is worth noting that the cytosolic neutral invertase tends to play a larger role in sink organs than does the vacuolar acid invertase. Studies on maize null mutants of the cytosolic invertase (*Mn1*) had miniature seeds caused by arrested endosperm development (Miller and Chourey, 1992), whereas overexpression of *Mn1* increased grain yield and starch content (Li et al., 2013). Similar studies in rice, tomato, and cotton (*Gossypium hirsutum* L.) have also found consistent phenotypes with cytosolic neutral invertase (Wang et al., 2008; Zanor et al., 2009; Wang and Ruan, 2012). Other studies on vacuolar invertase inhibitors showed a significant reduction of cold-induced sweetening in potato tubers (via a reduction in sucrose accumulation in tubers) by restricting the activities of vacuolar acid invertase (McKenzie et al., 2013; Brummell et al., 2011). These studies suggest the importance of sucrose unloading to sink organs; hence vacuolar acid and cytosolic invertases are targets for post-translational regulation toward starch storage and DM accumulation (Tang et al., 2016).

The hexoses cleaved from sucrose are rapidly phosphorylated into hexose monophosphates by hexokinase and fructokinase (Junker, 2004; Ap Rees, 1992; Appeldoorn et al., 1997; Renz et al., 1993) and they proceed to the starch biosynthesis or glycolytic pathways. As shown in the central pathway in Fig. 3, the resulting hexose monophosphates (including glucose-1-phosphate, glucose-6-phosphate and fructose-6-phosphate) are interconverted by the enzymes phosphoglucose mutase and phosphoglucose isomerase (*Manes.18G060600*) (Junker, 2004). Phosphoglucose isomerase connects the Calvin cycle pathway with the starch biosynthetic pathway in illuminated plant leaves (Bahaji et al., 2015). It also plays a key role in the glycolytic pathway and in the regeneration of glucose-6-phosphate in the oxidative pentose pathway in heterotrophic organs and nonilluminated plant leaves (Bahaji et al., 2015). It is strongly inhibited by light (Heuer et al., 1982) and by an intermediate Calvin cycle molecule, 3-phosphoglycerate (Dietz, 1985), which accumulates in the chloroplast during illumination and allosterically activates AGPase (Kleczkowski, 1999, 2000). The second phosphorylation step in the glycolytic pathway is the phosphorylation of fructose-6-phosphate to fructose-1,6-bisphosphate by phosphofructokinase (*Manes.09G077800*). Interestingly, transgenic studies overexpressing 6-phosphofructokinase in potato found no changes in the transgenic tuber phenotype compared with the controls but had an increased flux of cytosolic 3-phosphoglycerate that did not affect the amount of starch that accumulated in the tubers (Sweetlove et al., 2001; Burrell et al., 1994). It is noteworthy that our RHM results identified a signal on chromosome 9 in both yellow and white cassava that corresponds to the position of a phosphofructokinase in cassava.

Fructose-bisphosphate aldolase (FDA), a candidate from the Calvin cycle pathway (*Manes.04G007900*), is known to play a key role in carbohydrate biosynthesis. Changes in FDA activity have marked consequences for photosynthesis, C partitioning, growth, yield, and improved uniformity of solids in potato and other plants (Haake et al., 1998; Barry et al., 2002). Transgenic plants [including potato, maize, rice, canola (*Brassica napus* L.), and other crops] that expressed the *Escherichia coli* FDA gene in their chloroplasts had significantly higher root mass, leaf phenotypes with significantly higher starch accumulation, and lower leaf sucrose than control plants expressing the null vector (Barry et al., 2002).

### Implications for the Breeding of High DM White Cassava Varieties or High-DM, High-_β_ Carotene Yellow Cassava Varieties

The RHM results presented in this study suggest that DM content is under complex genetic control, particularly in the white cassava population. A network of genes and transcriptional regulons that are at the heart of sugar and starch biosynthesis were positionally associated with significant RHM regions in white and yellow cassava populations. The ‘hide a SNP’ analysis performed to validate the RHM results indicated that spurious associations caused by linkage may have been avoided in the RHM analysis even when large segments were involved (Fig. 2B). Given the genetic complexity of the cassava DM trait, we suggest that candidate genes, including invertases (neutral and acid) and FDA, may be targeted for gene editing or transgenic techniques to substantiate the role of these genes in DM and starch accumulation in cassava and to provide a clear path for their use in cassava breeding programs.

Dry matter content must work together with FYLD to make cassava production profitable and provide value for farmers and processors. To investigate whether some of the genes and gene families identified in the RHM analysis are also involved in the biological processes that lead to cassava FYLD, we validated their effects on FYLD using the same validation procedures and populations as above. The results showed prediction accuracies of 0.03 (SD = 0.02) for SnRKs on FYLD, 0.02 (SD = 0.02) for 53 likely candidates, 0.006 (SD = 0.03) for 53 unlikely candidates, 0.03 (SD = 0.02) for RHM-region genes, and -0.009 (SD = 0.02)for cassava starch pathway genes. These results suggest no single biological pathway that controls DM and FYLD. This is not surprising, since there is little genetic correlation between DM and FYLD (Kawano et al., 1987). It appears from the negative correlation between carotenoid content in roots and DM content in African cassava germplasm (Esuma et al., 2016; Akinwale et al., 2010) and from the link between bHLH and sugar biosynthesis (Krügel et al., 2012) that yellow flesh color is associated with the accumulation of reducing sugars in edible roots (Eleazu and Eleazu, 2012). This poses a more complex challenge for improving DM in African yellow cassava and shifts attention toward finding recombinant yellow cassava progenies that have high DM. Ceballos et al. (2015) states that the search for the appropriate recombinant is difficult in cassava breeding and advocates for the use of inbred progenitors when breeding for hybrid cassava.

**Fig. 4:**
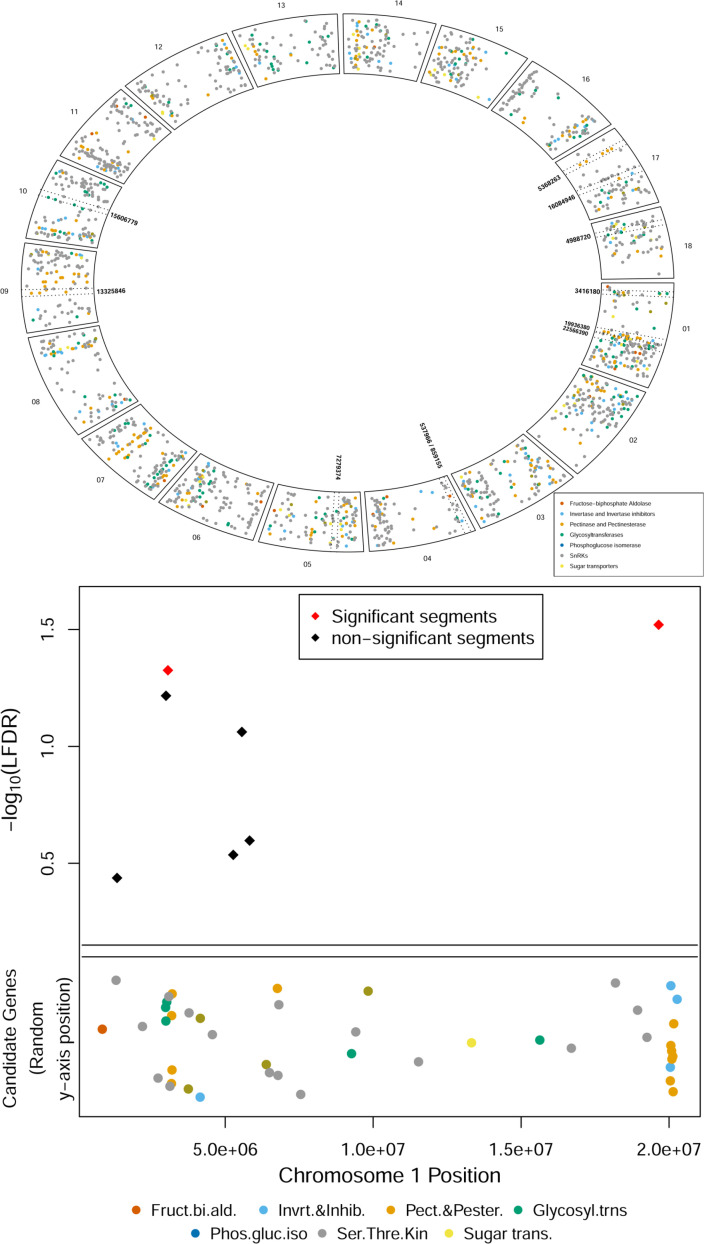
(A) Selected candidate genes of cassava and positions of significant regional heritability mapping (RHM) segments. Circos plots of carbohydrate biosynthesis candidate genes or gene families and significant RHM segments shown by paired dotted lines. Points are randomly scattered along the *y*-axis to avoid overlaps and visualize gene families better. (B) Zoom-in plot of candidate genes and significant RHM segments in a 21 Mb region of chromosome 1. The same genes or gene families as in (A) are shown along with two significant RHM segments. The double line separates candidate genes with random *y*-axis positions from–log_10_ (local false discovery rate) plotting of the significance of RHM segments.

In this paper, we have used candidate gene analysis in an attempt to understand the function of the genes or gene families positionally associated with the RHM hits. We do not make the claim that these candidates are causal genes detected by the RHM hits but rather we have shown, by using prediction accuracies, that these RHM hit loci were positionally associated with the DM trait in cassava (Fig. 1 and Fig.4A,B) thus resulting in better predictability than if random genes were used as controls. To validate the hypotheses presented in this paper regarding the candidate genes underlying DM accumulation in cassava, and to elucidate the physiological mechanisms involved in the expression of the DM trait in both yellow and white cassava, we recommend the use of genome editing or transgenic technology, and in-depth analysis of sugars and carbohydrates in cassava roots, stems, and leaves. Similar studies in potato have benefited and informed potato breeding, and the same will be true of cassava as new insights become available.

## Conclusion

By using RHM analysis, we demonstrate the complex genetic architecture of DM content in high-DM white African cassava. Candidate gene analysis revealed the possible roles of SnRKs, vacuolar and neutral invertases, phosphoglucose isomerase, and FDA in the regulation of sugar and starch biosynthesis in cassava. The RHM analysis indicated that inheritance of DM content in the high-DM white cassava population is more polygenic than that in the low-DM yellow cassava population. We examined the utility of models based on the genome-wide candidate genes found in this study with prediction accuracies in a different but related population and found appreciable predictive ability compared with what is obtained when whole-genome markers were used. Transcriptional regulators such as bHLH may be involved in flesh root color and sugar biosynthesis in cassava, as shown in potato. We recommend further studies using genome editing or transgenic technology to better understand these mechanisms and to inform and accelerate breeding efforts for cassava.

## Supplemental Information

Supplemental Table S1. Phytozome IDs and other genomic information about genes annotated as members of the SnRK family used for the validation of SnRKs in this study.

## References

[cit0001] Adewusi, S.R., and J.H. Bradbury 1993 Carotenoids in cassava: Comparison of open‐column and HPLC methods of analysis. J. Sci. Food Agric. 62(4):375–383. doi:10.1002/jsfa.2740620411

[cit0002] Akdemir, D., and U.G. Okeke 2014 EMMREML: Fitting mixed models with known covariance structures. R package version 2.0. https://cran.r-project.org/web/packages/EMMREML/index.html (accessed 29 Sept. 2017).

[cit0003] Akinwale, M.G., R.D. Aladesanwa, B.O. Akinyele, A.G.O. Dixon, and A.C. Odiyi 2010 Inheritance of β-carotene in cassava (Manihot esculenta Crantz). Int. J. Genet. Mol. Biol. 2(10):198–201

[cit0004] Aniedu, C. and Omodamiro, R.M, 2012 Use of newly bred β-carotene cassava in production of value-added products: Implication for food security in Nigeria. GJSFR, 12(10-D):11–16

[cit0005] Ap Rees, T 1992 Synthesis of storage starch. In: C.J. Pollock, J.F. Farrar, A.J. Gordon, editors, Carbon partitioning within and between organisms. Bios Scientific Publishers, Oxford, UK p. 115–131

[cit0006] Appeldoorn, N.J., S.M. de Bruijn, E.A. Koot-Gronsveld, R.G. Visser, D. Vreugdenhil, and L.H. van der Plas 1997 Developmental changes of enzymes involved in conversion of sucrose to hexose-phosphate during early tuberisation of potato. Planta 202(2):220–226. doi:10.1007/s004250050122

[cit0007] Atmodjo, M.A., Y. Sakuragi, X. Zhu, A.J. Burrell, S.S. Mohanty, J.A. Atwood, et al 2011 Galacturonosyltransferase (GAUT) 1 and GAUT7 are the core of a plant cell wall pectin biosynthetic homogal acturonan:galacturonosyltransferase complex. Proc. Natl. Acad. Sci. USA 108(50):20225–20230. doi:10.1073/pnas.1112816108 22135470 PMC3250160

[cit0008] Ayres, D.L., A. Darling, D.J. Zwickl, P. Beerli, M.T. Holder, P.O. Lewis, et al 2011 BEAGLE: An application programming interface and high-performance computing library for statistical phylogenetics. Syst. Biol. 61(1):170–173. doi:10.1093/sysbio/syr100. PMC3243739 21963610

[cit0009] Bahaji, A., Á.M. Sánchez-López, N. De Diego, F.J. Muñoz, E. Baroja-Fernández, J. Li, et al 2015 Plastidic phosphoglucose isomerase is an important determinant of starch accumulation in mesophyll cells, growth, photosynthetic capacity, and biosynthesis of plastidic cytokinins in Arabidopsis. PLoS One 10(3):e0119641. doi:10.1371/journal.pone.0119641 25811607 PMC4374969

[cit0010] Barrios, E.A., and R. Bressani 1967 Composicion quimica de la raiz y de la hoja de algunas variedades de yuca Manihot. Turrialba 17:314–320

[cit0011] Barry, G.F., N. Cheikh, and G.M. Kishore 2002 Expression of fructose 1,6 bisphosphate aldolase in transgenic plants. U.S. Patent 6441,277. Date issued: 16 Dec. 2003.

[cit0012] Bodmer, W., and I. Tomlinson 2010 Rare genetic variants and the risk of cancer. Curr. Opin. Genet. Dev. 20(3):262–267. doi:10.1016/j.gde.2010.04.016 20554195

[cit0013] Bouis, H.E., C. Hotz, B. McClafferty, J.V. Meenakshi, and W.H. Pfeiffer 2011 Biofortification: A new tool to reduce micronutrient malnutrition. Food Nutr. Bull. 32(1):S31–S40. doi:10.1177/15648265110321S105 21717916

[cit0014] Brummell, D.A., R.K. Chen, J.C. Harris, H. Zhang, C. Hamiaux, A.V. Kralicek, et al 2011 Induction of vacuolar invertase inhibitor mRNA in potato tubers contributes to cold-induced sweetening resistance and includes spliced hybrid mRNA variants. J. Exp. Bot. 62(10):3519–3534. doi:10.1093/jxb/err043 21393382 PMC3130176

[cit0015] Burrell, M.M., P.J. Mooney, M. Blundy, D. Carter, F. Wilson, J. Green, et al 1994 Genetic manipulation of 6-phosphofructokinase in potato tubers. Planta 194(1):95–101. doi:10.1007/BF00201039

[cit0016] Caballero, A., A. Tenesa, and P.D. Keightley 2015 The nature of genetic variation for complex traits revealed by GWAS and regional heritability mapping analyses. Genetics 201(4):1601–1613. doi:10.1534/genetics.115.177220 26482794 PMC4676519

[cit0017] Cantarel, B.L., P.M. Coutinho, C. Rancurel, T. Bernard, V. Lombard, and B. Henrissat 2009 The Carbohydrate-Active EnZymes database (CAZy): An expert resource for glycogenomics. Nucleic Acids Res. 37(Suppl 1):D233–D238. doi:10.1093/nar/gkn663 18838391 PMC2686590

[cit0018] Ceballos, H., C.A. Iglesias, J.C. Pérez, and A.G. Dixon 2004 Cassava breeding: Opportunities and challenges. Plant Mol. Biol. 56(4):503–516. doi:10.1007/s11103-004-5010-5 15630615

[cit0019] Ceballos, H., R.S. Kawuki, V.E. Gracen, G.C. Yencho, and C.H. Hershey 2015 Conventional breeding, marker-assisted selection, genomic selection and inbreeding in clonally propagated crops: A case study for cassava. Theor. Appl. Genet. 128(9):1647–1667 26093610 10.1007/s00122-015-2555-4 PMC4540783

[cit0020] Ceballos, H., N. Morante, T. Sánchez, D. Ortiz, I. Aragon, A.L. Chávez, et al 2013 Rapid cycling recurrent selection for increased carotenoids content in cassava roots. Crop Sci. 53(6):2342–2351. doi:10.2135/cropsci2013.02.0123

[cit0021] Chávez, A.L., T. Sánchez, G. Jaramillo, J. Bedoya, J. Echeverry, E.A. Bolaños, et al 2005 Variation of quality traits in cassava roots evaluated in landraces and improved clones. Euphytica 143(1-2):125–133. doi:10.1007/s10681-005-3057-2

[cit0022] Chincinska, I.A., J. Liesche, U. Krügel, J. Michalska, P. Geigenberger, B. Grimm, et al 2008 Sucrose transporter StSUT4 from potato affects flowering, tuberization, and shade avoidance response. Plant Physiol. 146(2):515–528. doi:10.1104/pp.107.112334 18083796 PMC2245842

[cit0023] Crozet, P., L. Margalha, A. Confraria, A. Rodrigues, C. Martinho, M. Adamo, et al 2014 Mechanisms of regulation of SNF1/AMPK/SnRK1 protein kinases. Front. Plant Sci. 5:190. doi:10.3389/fpls.2014.00190 24904600 PMC4033248

[cit0024] de Godoy, F., L. Bermúdez, B.S. Lira, A.P. de Souza, P. Elbl, D. Demarco, et al 2013 Galacturonosyltransferase 4 silencing alters pectin composition and carbon partitioning in tomato. J. Exp. Bot. 64(8):2449–2466. doi:10.1093/jxb/ert106 23599271 PMC3654432

[cit0025] De Jong, W.S., N.T. Eannetta, D.M. De Jong, and M. Bodis 2004 Candidate gene analysis of anthocyanin pigmentation loci in the Solanaceae. Theor. Appl. Genet. 108(3):423–432. doi:10.1007/s00122-003-1455-1 14523517

[cit0026] Dietz, K.J 1985 A possible rate-limiting function of chloroplast hexosemonophosphate isomerase in starch synthesis of leaves. Biochim. Biophys. Acta 839(3):240–248. doi:10.1016/0304-4165(85)90004-2

[cit0027] Du, J., H. Yin, S. Zhang, Z. Wei, B. Zhao, J. Zhang, et al 2012 Somatic embryogenesis receptor kinases control root development mainly via brassinosteroid‐independent actions in Arabidopsis thaliana. J. Integr. Plant Biol. 54(6):388–399. doi:10.1111/j.1744-7909.2012.01124.x 22525267

[cit0028] Eleazu, C.O., and K.C. Eleazu 2012 Determination of the proximate composition, total carotenoid, reducing sugars and residual cyanide levels of flours of 6 new yellow and white cassava (Manihot esculenta Crantz) varieties. Am. J. Food Technol. 7(10):642–649. doi:10.3923/ajft.2012.642.649

[cit0029] El-Sharkawy, M.A 2003 Cassava biology and physiology. Plant Mol. Biol. 53(5):621–641. doi:10.1023/B:PLAN.0000019109.01740.c6 15669146

[cit0030] Elshire, R.J., J.C. Glaubitz, Q. Sun, J.A. Poland, K. Kawamoto, E.S. Buckler, et al 2011 A robust, simple genotyping-by-sequencing (GBS) approach for high diversity species. PLoS One 6(5):e19379. doi:10.1371/journal.pone.0019379 21573248 PMC3087801

[cit0031] Enidiok, S.E., L.E. Attah, and C.A. Otuechere 2008 Evaluation of moisture, total cyanide and fiber contents of garri produced from cassava (Manihot utilissima) varieties obtained from Awassa in Southern Ethiopia. Pak. J. Nutr. 7:625–629. doi:10.3923/pjn.2008.625.629

[cit0032] Esuma, W., R.S. Kawuki, L. Herselman, and M.T. Labuschagne 2016 Diallel analysis of provitamin A carotenoid and dry matter content in cassava (Manihot esculenta Crantz). Breed. Sci. 66(4):627–635. doi:10.1270/jsbbs.15159 PMC5010302 27795688

[cit0033] Food and Agricultural Organization of the United Nations 2013 FAOSTAT statistics database. Food and Agriculture Organization of the United Nations, Rome.

[cit0034] Geigenberger, P 2003 Regulation of sucrose to starch conversion in growing potato tubers. J. Exp. Bot. 54(382):457–465. doi:10.1093/jxb/erg074 12508056

[cit0035] Gibson, G 2012 Rare and common variants: Twenty arguments. Nat. Rev. Genet. 13(2):135–145. doi:10.1038/nrg3118 22251874 PMC4408201

[cit0036] Glaubitz, J., T. Casstevens, R. Elshire, J. Harriman, and E.S. Buckler 2012 TASSEL 3.0 genotyping by sequencing (GBS) pipeline documentation. USDA-ARS. http://www.maizegenetics.net/tassel/docs/Tassel-PipelineGBS.pdf (accessed 29 Sept. 2017) 10.1371/journal.pone.0090346 PMC3938676 24587335

[cit0037] Goodstein, D.M., Shu, S., Howson, R., Neupane, R., Hayes, R.D., Fazo, J., et al 2012 Phytozome: A comparative platform for green plant genomics. Nucleic Acids Res. 40(D1), D1178–D1186. doi:10.1093/nar/gkr944 22110026 PMC3245001

[cit0038] Haake, V., R. Zrenner, U. Sonnewald, and M. Stitt 1998 A moderate decrease of plastid aldolase activity inhibits photosynthesis, alters the levels of sugars and starch, and inhibits growth of potato plants. Plant J. 14(2):147–157. doi:10.1046/j.1365-313X.1998.00089.x 9628012

[cit0039] Halford, N.G., and D.G. Hardie 1998 SNF1-related protein kinases: Global regulators of carbon metabolism in plants? Plant Mol. Biol. 37(5):735–748. doi:10.1023/A:1006024231305 9678569

[cit0040] Halford, N.G., S. Hey, D. Jhurreea, S. Laurie, R.S. McKibbin, M. Paul, et al 2003 Metabolic signalling and carbon partitioning: Role of Snf1‐related (SnRK1) protein kinase. J. Exp. Bot. 54(382):467–475. doi:10.1093/jxb/erg038 12508057

[cit0041] HarvestPlus 2009 Provitamin A cassava. HarvestPlus. http://webarchive.nationalarchives.gov.uk/20100404130540/ http://www.research4development.info/PDF/Outputs/Misc_Crop/HarvstPlus_Cassava_Strategy.pdf (accessed 25 Sept. 2017).

[cit0042] Heuer, B., M.J. Hansen, and L.E. Anderson 1982 Light modulation of phosphofructokinase in pea leaf chloroplasts. Plant Physiol. 69(6):1404–1406. doi:10.1104/pp.69.6.1404 16662412 PMC426427

[cit0043] Holleman, L.W.J., and A. Aten 1956 Processing of cassava and cassava products in rural industries. FAO Agricultural Developed Paper 54. Food and Agricultural Organization of the United Nations, Rome.

[cit0044] Iglesias, C.A., and C.H. Hershey 1991 True cassava seed: Research for a production alternative. Acta Hortic. 380: 164–171. doi:10.17660/ActaHortic.1994.380.24

[cit0045] Iglesias, C., Hershey, C., Calle, F., and Bolaños, A 1994 Propagating cassava (Manihot esculenta) by sexual seed. Exp. Agric. 30(3):283–290.

[cit0046] Jossier, M., J.P. Bouly, P. Meimoun, A. Arjmand, P. Lessard, S. Hawley, et al 2009 SnRK1 (SNF1‐related kinase 1) has a central role in sugar and ABA signalling in Arabidopsis thaliana. Plant J. 59(2):316–328. doi:10.1111/j.1365-313X.2009.03871.x 19302419

[cit0047] Junker, B.H, 2004 Sucrose breakdown in the potato tuber. Mathematisch-Naturwissenschaftliche Fakultät. Universität Potsdam, Postsdam-Golm, Brandenburg, Germany.

[cit0048] Kawano, K., W.M.G. Fukuda, and U. Cenpukdee 1987 Genetic and environmental effects on dry matter content of cassava root. Crop Sci. 27(1):69–74. doi:10.2135/cropsci1987.0011183X002700010018x

[cit0049] Keating, B.A., G.L. Wilson, and J.P. Evenson 1988 Effects of length, thickness, orientation, and planting density of cassava (Manihot esculenta Crantz) planting material on subsequent establishment, growth and yield. E. Afr. Agric. For. J 53:145–149

[cit0050] Kimura, M., C.N. Kobori, D.B. Rodriguez-Amaya, and P. Nestel 2007 Screening and HPLC methods for carotenoids in sweetpotato, cassava and maize for plant breeding trials. Food Chem. 100(4):1734–1746. doi:10.1016/j.foodchem.2005.10.020

[cit0051] Kleczkowski, L.A 1999 A phosphoglycerate to inorganic phosphate ratio is the major factor in controlling starch levels in chloroplasts via ADP‐glucose pyrophosphorylase regulation. FEBS Lett. 448(1):153–156. doi:10.1016/S0014-5793(99)00346-4 10217430

[cit0052] Kleczkowski, L.A 2000 Is leaf ADP-glucose pyrophosphorylase an allosteric enzyme? Biochim. Biophys. Acta 1476(1):103–108. doi:10.1016/S0167-4838(99)00229-0 10606772

[cit0053] Krügel, U., H.X. He, K. Gier, J. Reins, I. Chincinska, B. Grimm, et al 2012 The potato sucrose transporter StSUT1 interacts with a DRM-associated protein disulfide isomerase. Mol. Plant 5(1):43–62. doi:10.1093/mp/ssr048 21746698

[cit0054] La Frano, M.R., L.R. Woodhouse, D.J. Burnett, and B.J. Burri 2013 Biofortified cassava increases β-carotene and vitamin A concentrations in the TAG-rich plasma layer of American women. Br. J. Nutr. 110(02):310–320. doi:10.1017/S0007114512005004 23332040

[cit0055] Laurie, S., R.S. McKibbin, and N.G. Halford 2003 Antisense SNF1-related (SnRK1) protein kinase gene represses transient activity of an α‐amylase (α‐Amy2) gene promoter in cultured wheat embryos. J. Exp. Bot. 54(383):739–747. doi:10.1093/jxb/erg085 12554717

[cit0056] Li, B., H. Liu, Y. Zhang, T. Kang, L. Zhang, J. Tong, et al 2013 Constitutive expression of cell wall invertase genes increases grain yield and starch content in maize. Plant Biotechnol. J. 11(9):1080–1091. 23926950 10.1111/pbi.12102

[cit0057] Lim, H.K 1968 Composition data of feeds and concentrates. Malay. Agric. J. 46:63–79.

[cit0058] Lin, Y., T. Liu, J. Liu, X. Liu, Y. Ou, H. Zhang, et al 2015 Subtle regulation of potato acid invertase activity by a protein complex of invertase, invertase inhibitor, and SUCROSE NONFERMENTING1-RELATED PROTEIN KINASE. Plant Physiol. 168(4):1807–1819. doi:10.1104/pp.15.00664 26134163 PMC4528764

[cit0059] Liu, W., Zhou, Y., Sanchez, T., Ceballos, H. and White, W.S, 2010 The vitamin A equivalence of β-carotene in β-carotene-biofortified cassava ingested by women. FASEB J. 24(1):92–97.

[cit0060] Ly, D., M. Hamblin, I. Rabbid, G. Melakud, M. Bakared, H.G. Gauch Jr, et al. 2013 Relatedness and genotype× environment interaction affect prediction accuracies in genomic selection: A study in cassava. Crop Sci. 53(4):1312–1325. doi:10.2135/cropsci2012.11.0653

[cit0061] Maass, D., J. Arango, F. Wüst, P. Beyer, and R. Welsch 2009 Carotenoid crystal formation in Arabidopsis and carrot roots caused by increased phytoene synthase protein levels. PLoS One 4(7):e6373. doi:10.1371/journal.pone.0006373 19636414 PMC2712097

[cit0062] Maziya-Dixon, B., A.G.O. Dixon, and A.-R.A. Adebowale 2007 Targeting different end uses of cassava: Genotypic variations for cyanogenic potentials and pasting properties. Int. J. Food Sci. Technol. 42:969–976. doi:10.1111/j.1365-2621.2006.01319.x

[cit0063] McKenzie, M.J., R.K. Chen, J.C. Harris, M.J. Ashworth, and D.A. Brummell 2013 Post‐translational regulation of acid invertase activity by vacuolar invertase inhibitor affects resistance to cold‐induced sweetening of potato tubers. Plant Cell Environ. 36(1):176–185. doi:10.1111/j.1365-3040.2012.02565.x 22734927

[cit0064] McKibbin, R.S., N. Muttucumaru, M.J. Paul, S.J. Powers, M.M. Burrell, S. Coates, et al 2006 Production of high-starch, low-glucose potatoes through over-expression of the metabolic regulator SnRK1. Plant Biotechnol. J. 4(4):409–418. doi:10.1111/j.1467-7652.2006.00190.x 17177806

[cit0065] Meenakshi, J.V., A. Banerji, V. Manyong, K. Tomlins, P. Hamukwala, R. Zulu, et al 2010 Consumer acceptance of provitamin A orange maize in rural Zambia HarvestPlus Working Paper No. 4. HarvestPlus, Washington, DC.

[cit0066] Meenakshi, J.V., N. Johnson, M. Victor, H. De Groote , J. Javelosa , D. Yanggen, et al. 2007 How cost-effective is biofortification in combating micronutrient malnutrition? An ex ante assessment. World Dev, 38(1):64–75.

[cit0067] Miller, M.E., and P.S. Chourey 1992 The maize invertase-deficient miniature-1 seed mutation is associated with aberrant pedicel and endosperm development. Plant Cell 4(3):297–305. doi:10.1105/tpc.4.3.297 12297647 PMC160130

[cit0068] Momma, M., and Z. Fujimoto 2012 Interdomain disulfide bridge in the rice granule bound starch synthase I catalytic domain as elucidated by X-ray structure analysis. Biosci. Biotechnol. Biochem. 76(8):1591–1595. 10.1271/bbb.120305 22878205

[cit0069] Nagamine, Y., R. Pong-Wong, P. Navarro, V. Vitart, C. Hayward, I. Rudan, et al. 2012 Localising loci underlying complex trait variation using regional genomic relationship mapping. PLoS One 7(10):e46501. doi:10.1371/journal.pone.0046501 23077511 PMC3471913

[cit0070] Okechukwu, R.U., and A.G.O. Dixon 2008 Genetic gains from 30 years of cassava breeding in Nigeria for storage root yield and disease resistance in elite cassava genotypes. J. Crop Improv. 22:181–208. doi:10.1080/15427520802212506

[cit0071] Pfeiffer, W.H. and McClafferty, B, 2007 HarvestPlus: Breeding crops for better nutrition. Crop Sci. 47(Suppl.3): S88–S105. doi:10.2135/cropsci2007.09.0020IPBS

[cit0072] Polge, C., and M. Thomas 2007 SNF1/AMPK/SnRK1 kinases, global regulators at the heart of energy control? Trends Plant Sci. 12(1):20–28. doi:10.1016/j.tplants.2006.11.005 17166759

[cit0073] Purcell, P.C., A.M. Smith, and N.G. Halford 1998 Antisense expression of a sucrose non‐fermenting‐1‐related protein kinase sequence in potato results in decreased expression of sucrose synthase in tubers and loss of sucrose‐inducibility of sucrose synthase transcripts in leaves. Plant J. 14(2):195–202. doi:10.1046/j.1365-313X.1998.00108.x

[cit0074] Quinlan, A.R., and I.M. Hall 2010 BEDTools: A flexible suite of utilities for comparing genomic features. Bioinformatics 26(6):841–842. doi:10.1093/bioinformatics/btq033 20110278 PMC2832824

[cit0075] R Development Core Team 2016 R: A language and environment for statistical computing. R Foundation for Statistical Computing, http://www.R-project.org (accessed 29 Sept. 2017).

[cit0076] Radchuk, R., R.J. Emery, D. Weier, H. Vigeolas, P. Geigenberger, J.E. Lunn, et al. 2010 Sucrose non‐fermenting kinase 1 (SnRK1) coordinates metabolic and hormonal signals during pea cotyledon growth and differentiation. Plant J. 61(2):324–338. doi:10.1111/j.1365-313X.2009.04057.x 19845880

[cit0077] Radchuk, R., V. Radchuk, W. Weschke, L. Borisjuk, and H. Weber 2006 Repressing the expression of the SUCROSE NONFERMENTING-1-RELATED PROTEIN KINASE gene in pea embryo causes pleiotropic defects of maturation similar to an abscisic acid-insensitive phenotype. Plant Physiol. 140(1):263–278. doi:10.1104/pp.105.071167 16361518 PMC1326049

[cit0078] Raji, A.A., A.G.O. Dixon, and T.A.O. Ladeinde 2007 Agronomic traits and tuber quality attributes of farmer grown cassava (Manihot esculenta) landraces in Nigeria. J. Trop. Agric. 45:9–13.

[cit0079] Renz, A., L. Merlo, and M. Stitt 1993 Partial purification from potato tubers of three fructokinases and three hexokinases which show differing organ and developmental specificity. Planta 190(2):156–165.

[cit0080] Resende, R.T., M.D.V. Resende, F.F. Silva, C.F. Azevedo, E.K. Takahashi, O.B. Silva‐Junior, et al. 2017 Regional heritability mapping and genome‐wide association identify loci for complex growth, wood and disease resistance traits in Eucalyptus. New Phytol. 213(3):1287–1300. doi:10.1111/nph.14266 28079935

[cit0081] Riggio, V., and R. Pong-Wong 2014 Regional heritability mapping to identify loci underlying genetic variation of complex traits. BMC Proc. 8(5):S3. doi:10.1186/1753-6561-8-S5-S3 PMC4195407 25519517

[cit0082] Riggio, V., O. Matika, R. Pong-Wong, M.J. Stear, and S.C. Bishop 2013 Genome-wide association and regional heritability mapping to identify loci underlying variation in nematode resistance and body weight in Scottish Blackface lambs. Heredity 110(5):420–429. doi:10.1038/hdy.2012.90 23512009 PMC3630823

[cit0083] Rolland, F., B. Moore, and J. Sheen 2002 Sugar sensing and signaling in plants. Plant Cell 14(Suppl 1):S185–S205. 12045277 10.1105/tpc.010455 PMC151255

[cit0084] Safo‐Kantanka, O., and J. Owusu‐Nipah 1992 Cassava varietal screening for cooking quality: Relationship between dry matter, starch content, mealiness and certain microscopic observations of the raw and cooked tuber. J. Sci. Food Agric. 60(1):99–104. doi:10.1002/jsfa.2740600116

[cit0085] Saithong, T., O. Rongsirikul, S. Kalapanulak, P. Chiewchankaset, W. Siriwat, S. Netrphan, et al 2013 Starch biosynthesis in cassava: A genome-based pathway reconstruction and its exploitation in data integration. BMC Syst. Biol. 7(1):75. doi:10.1186/1752-0509-7-75 23938102 PMC3847483

[cit0086] Shirali, M., R. Pong-Wong, P. Navarro, S. Knott, C. Hayward, V. Vitart, et al. 2015 Regional heritability mapping method helps explain missing heritability of blood lipid traits in isolated populations. Heredity. 116(3):333–338. doi:10.1038/hdy.2015.107 26696135 PMC4751621

[cit0087] Story, J.D., A.J. Bass, A. Dabney, and D. Robinson 2015 qvalue: Q-value estimation for false discovery rate control. R package version 2.2.2. GitHub Inc. http://github.com/jdstorey/qvalue (accessed 29 Sept. 2017).

[cit0088] Storey, J.D., and R. Tibshirani 2003 Statistical significance for genome-wide experiments. Proc. Natl. Acad. Sci. USA 100:9440–9445. doi:10.1073/pnas.1530509100 12883005 PMC170937

[cit0089] Ssemakula, G., A.G.O. Dixon, and B. Maziya-Dixon 2007 Stability of total carotenoid concentration and fresh yield of selected yellow-fleshed cassava (Manihot esculenta Crantz). J. Trop. Agric. 45(1-2):14–20.

[cit0090] Sweetlove, L.J., N.J. Kruger, and S.A. Hill 2001 Starch synthesis in transgenic potato tubers with increased 3-phosphoglyceric acid content as a consequence of increased 6-phosphofructokinase activity. Planta 213(3):478–482. doi:10.1007/s004250100544 11506372

[cit0091] Tai, H.H., C. Goyer, and A.M. Murphy 2013 Potato MYB and bHLH transcription factors associated with anthocyanin intensity and common scab resistance. Botany 91(10):722–730. doi:10.1139/cjb-2012-0025

[cit0092] Takatsuji, H 1998 Zinc-finger transcription factors in plants. Cell. Mol. Life Sci. 54(6):582–596. doi:10.1007/s000180050186 9676577 PMC11147231

[cit0093] Takatsuji, H 1999 Zinc-finger proteins: The classical zinc finger emerges in contemporary plant science. Plant Mol. Biol. 39(6):1073–1078. doi:10.1023/A:1006184519697 10380795

[cit0094] Tanaka, M., Y. Takahata, H. Nakayama, M. Nakatani, and M. Tahara 2009 Altered carbohydrate metabolism in the storage roots of sweetpotato plants overexpressing the SRF1 gene, which encodes a Dof zinc finger transcription factor. Planta 230(4):737–746. doi:10.1007/s00425-009-0979-2 19618208

[cit0095] Tang, X., T. Su, M. Han, L. Wei, W. Wang, Z. Yu, et al 2016 Suppression of extracellular invertase inhibitor gene expression improves seed weight in soybean (Glycine max). J. Exp. Bot. 68(3):469–482. doi:10.1093/jxb/erw425 PMC5441900 28204559

[cit0096] Tiessen, A., K. Prescha, A. Branscheid, N. Palacios, R. McKibbin, N.G. Halford, et al 2003 Evidence that SNF1‐related kinase and hexokinase are involved in separate sugar‐signalling pathways modulating post‐translational redox activation of ADP‐glucose pyrophosphorylase in potato tubers. Plant J. 35(4):490–500. doi:10.1046/j.1365-313X.2003.01823.x 12904211

[cit0097] Toledo-Ortiz, G., E. Huq, and M. Rodríguez-Concepción 2010 Direct regulation of phytoene synthase gene expression and carotenoid biosynthesis by phytochrome-interacting factors. Proc. Natl. Acad. Sci. USA 107(25):11626–11631. doi:10.1073/pnas.0914428107 20534526 PMC2895139

[cit0098] Uemoto, Y., R. Pong-Wong, P. Navarro, V. Vitart, C. Hayward, J.F. Wilson, et al 2013 The power of regional heritability analysis for rare and common variant detection: Simulations and application to eye biometrical traits. Front. Genet. 4:232. doi:10.3389/fgene.2013.00232 24312116 PMC3832942

[cit0099] VanRaden, P.M 2008 Efficient methods to compute genomic predictions. J. Dairy Sci. 91:4414–4423. doi:10.3168/jds.2007-0980 18946147

[cit0100] Vimala, B., Nambisan, B., Theshara, R. and Unnikrishnam, M, 2009 Variability of carotenoids in yellow-flesh cassava (Manihot esculenta Crantz).Gene Conserve. 8(31):676–685.

[cit0101] Wang, E., J. Wang, X. Zhu, W. Hao, L. Wang, Q. Li, et al 2008 Control of rice grain-filling and yield by a gene with a potential signature of domestication. Nat. Genet. 40(11):1370–1374. doi:10.1038/ng.220 18820698

[cit0102] Wang, L., and Y.L. Ruan 2012 New insights into roles of cell wall invertase in early seed development revealed by comprehensive spatial and temporal expression patterns of GhCWIN1 in cotton. Plant Physiol. 160(2):777–787. doi:10.1104/pp.112.203893 22864582 PMC3461555

[cit0103] Weschke, W., R. Panitz, S. Gubatz, Q. Wang, R. Radchuk, H. Weber, et al 2003 The role of invertases and hexose transporters in controlling sugar ratios in maternal and filial tissues of barley caryopses during early development. Plant J. 33(2):395–411. doi:10.1046/j.1365-313X.2003.01633.x 12535352

[cit0104] Weschke, W., R. Panitz, N. Sauer, Q. Wang, B. Neubohn, H. Weber, et al 2000 Sucrose transport into barley seeds: Molecular characterization of two transporters and implications for seed development and starch accumulation. Plant J. 21(5):455–467. 10758497 10.1046/j.1365-313x.2000.00695.x

[cit0105] Wolfe, M.D., I.Y. Rabbi, C. Egesi, M. Hamblin, R. Kawuki, P. Kulakow, et al 2016 Genome-wide association and prediction reveals genetic architecture of cassava mosaic disease resistance and prospects for rapid genetic improvement. Plant Genome 9(2). doi:10.3835/plantgenome2015.11.0118 27898832

[cit0106] Wood, A.R., T. Esko, J. Yang, S. Vedantam, T.H. Pers, S. Gustafsson, et al 2014 Defining the role of common variation in the genomic and biological architecture of adult human height. Nat. Genet. 46(11):1173–1186. 25282103 10.1038/ng.3097 PMC4250049

[cit0107] Xue-Fei, D., C. Na, W. Li, Z. Xiao-Cui, Q. Bo, L. Tian-Lai, et al 2012 The SnRK protein kinase family and the function of SnRK1 protein kinase. Int. J. Agric. Biol. 14(4):575–579.

[cit0108] Yang, J., D. An, and P. Zhang 2011 Expression profiling of cassava storage roots reveals an active process of glycolysis/gluconeogenesis. J. Integr. Plant Biol. 53(3):193–211. doi:10.1111/j.1744-7909.2010.01018.x 21205184

[cit0109] Zanor, M.I., S. Osorio, A. Nunes-Nesi, F. Carrari, M. Lohse, B. Usadel, et al 2009 RNA interference of LIN5 in tomato confirms its role in controlling Brix content, uncovers the influence of sugars on the levels of fruit hormones, and demonstrates the importance of sucrose cleavage for normal fruit development and fertility. Plant Physiol. 150(3):1204–1218. doi:10.1104/pp.109.136598 19439574 PMC2705052

[cit0110] Zeng, Y., Navarro, P., Fernandez-Pujals, A.M., Hall, L.S., Clarke, T.K., Thomson, et al 2016 A combined pathway and regional heritability analysis indicates NETRIN1 pathway is associated with major depressive disorder. Biol. Psychiatry. 81(4):336–346. doi:10.1016/j.biopsych.2016.04.017 27422368 PMC5262437

[cit0111] Zhang, Y., C.S. Jung, and W.S. De Jong 2009 Genetic analysis of pigmented tuber flesh in potato. Theor. Appl. Genet. 119(1):143–150. doi:10.1007/s00122-009-1024-3 19363602 PMC2690854

